# Hormonal Regulation of Avocado (*Persea americana*) Across Altitudinal Gradients

**DOI:** 10.1002/pei3.70083

**Published:** 2025-09-08

**Authors:** Walid El Kayal, Maya Salameh, Diana Nacouzi

**Affiliations:** ^1^ Faculty of Agricultural and Food Sciences American University of Beirut Beirut Lebanon

**Keywords:** avocado, gene expression, maturity indices, phytohormones

## Abstract

Avocado ( *Persea americana* ) stands out as one of the most significant crops globally. Due to its abundance in essential nutrients and phytochemicals, its consumption and commercialization have notably surged in recent years. The interplay between genotype and environment profoundly influences fruit maturity dates and physicochemical attributes. This study aimed to assess the transcript levels of genes involved in hormone regulation and biosynthesis in avocado fruits, correlating their expression with the crop's physiological characteristics across varying altitudes during maturity and ripening stages. The study focused on five prevalent avocado varieties: Fuerte, Hass, Pinkerton, Lambhass, and Reed. Sixteen genes participating in diverse metabolic pathways and five hormones: Abscisic acid, Jasmonic acid, Salicylic acid, Gibberellic acid, and Zeatin were quantified over the harvesting season across seven locations in Lebanon. Results revealed a notable correlation between the expression of certain genes and hormone levels in the tested varieties, contingent upon both variety and location. Phytohormone quantification exhibited significant variations across locations compared to varieties. Additionally, physicochemical characteristics were evaluated, with principal component analysis demonstrating a positive correlation between some quantified phytohormones and maturity indices among varieties and locations. This study significantly advances our understanding of the intricate relationship among phytohormones, altitudes, fruit maturity, and ripening processes across five of the most common avocado varieties.

## 1 Introduction

**FIGURE 1:**
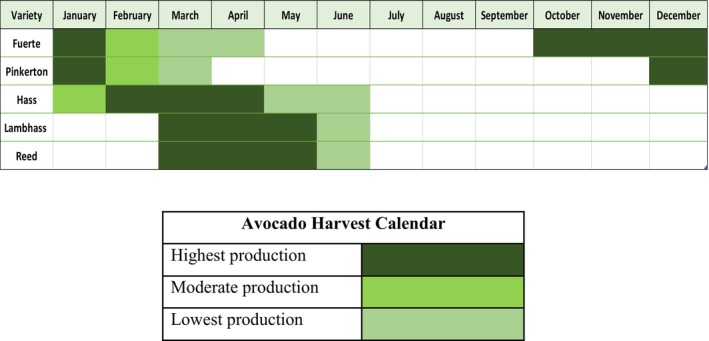
The harvesting calendar of avocado fruits in Lebanon (modified from Lebtrade, https://lebtrade.gov.lb/product/avocado/).

The cultivation of avocado ( *Persea americana* ) in Lebanon holds significant economic importance, with a planted area reaching 1580 ha in 2021. During the same year, 18,955 tons of avocado fruit were produced, with 8884 tons exported mainly to Europe and Gulf countries (Lebfresh 2024). The ripening season and harvesting calendar of the most common five varieties in the country (Fuerte, Pinkerton, Hass, Lambhass and Reed) are presented in Figure 1.

To compete with international exporting countries boasting higher production volumes, Lebanese avocado production must pivot towards offering a high‐quality product distinguished by attributes such as color, firmness, dry matter content, taste, softening rate, shelf‐life, and post‐harvest quality. A major challenge faced by the Lebanese avocado industry lies in standardizing fruit quality and ripening behavior amidst considerable variability in environmental conditions, soil types, and agronomic practices, all of which can influence ripening consistency. Various factors, including environmental conditions, soil management, agricultural practices, and fruit physiological variables, impact the ripening of avocado fruits (Rivera et al. 2017). While previous studies have identified commercial harvest indexes like dry matter, oil content, and fruit firmness as indicators to optimize harvest dates, these metrics alone may not suffice to determine a fruit's physiological age and subsequent ripening behavior (Rivera et al. 2017). Researchers have identified transcripts and hormone‐based biomarkers to describe the physiological age of avocado fruits, shedding light on variations in metabolic pathways and their regulation (Hernández et al. 2022).

Though research has primarily focused on the role of ethylene in avocado maturation and ripening (Hernández et al. 2022), the involvement of other phytohormones remains poorly understood. Suggestions have been made that avocado fruit ripening may be regulated not only by ethylene but also by other phytohormones (Vincent et al. 2020). Studies have described a complex hormonal interplay during avocado fruit maturation and ripening, implicating hormones such as abscisic acid (ABA), jasmonates, gibberellins, and auxins (Meyer et al. 2017; Vincent et al. 2020). The coordination of different plant hormones governs fruit growth and development, with hormones like auxins and gibberellins influencing genes involved in primary metabolism and cell division (Ruiu et al. 2015; Pedreschi et al. 2019). Moreover, recent research has highlighted the role of genes regulating cell wall structural proteins, such as homologs to proline‐rich proteins (PRPs), in avocado fruit firmness (Vergara‐Pulgar et al. 2019). Avocado fruits exhibit considerable variability and do not uniformly behave after harvest, necessitating the use of plant growth regulators (PGRs) to manage tree growth and yield in orchards (Lovatt 2008; Blakey et al. 2009).

To ensure profitability for farmers and consumer acceptance, it is crucial to study the actual maturity of avocado fruits before harvesting. Maturity characteristics can be assessed through parameters such as dry matter, oil content, fruit weight, firmness, caliber, and color. However, studying these indices can be challenging, often requiring expensive and time‐consuming laboratory equipment and personnel. To address this, there is a growing trend towards using faster and non‐destructive methods like diffuse reflection near‐infrared (NIR) spectroscopy.

This research aims to quantify and correlate phytohormones and physicochemical parameters to better understand the complex interplay that modulates avocado postharvest ripening. Given the significant impact of altitudes on phenology and fruit quality, the study was conducted across seven common locations where avocado varieties are grown, varying in altitudes from 158 m to 600 m. The supporting information Figure S1 represents a map of the seven locations: south (Nmeiriyeh, Marwanieh, Ansar, and Abbasiyeh) and north locations (Aadbel, Beit Mallat and Qloud El Barka) used in this study and their altitudes.

The data generated from this study could contribute to establishing a maturity prediction model for different avocado varieties, relying on phytohormone levels, physicochemical parameters, and geographical locations as key inputs.

## 2 Materials and Methods

### 2.1 Plant Material

Avocado fruits of different varieties were picked by hand from 6 different locations of south and north Lebanon (Nmeiriyeh, Merweniyeh, Kfar Hay, Ansar, Abasiyeh, Qloud El Barka and Markabta). The supporting information Table S1 presents variety name, location, and their GPS units (Longitude and Latitude). Each orchard was split into rows, and fruits were harvested from each row to ensure the representatives of the experiment; then, samples were sent to the laboratory on the same day for gene expression and hormone quantification analysis. Samples were collected from trees representing 10%–15% from every orchard. In other words, if an orchard has 300 trees, at least 30–40 trees and 6 fruits per tree were sampled to represent the varied locations of this orchard. The fruits were placed in perforated transparent bags and stored with ice until reaching the laboratory's fridge within a few hours. The avocado samples were visually inspected at the laboratory to ensure that they were not subjected to any damage during transportation, and if they were, the damaged ones were excluded from the samples.

### 2.2 Physicochemical Characteristics

The avocado fruits were subjected to several measurements. First, four fruits from each tree were individually weighed, and their circumference and length were measured using a caliper. Then, the firmness of each fruit was measured four times using a penetrometer on both sides without skin. Firmness was measured after peeling off the skin using an Effegi FT 011 (0–5 kg) penetrometer equipped with an 11‐mm plunger. Afterward, each fruit was divided into two parts. The first part was labeled and then either freeze‐dried for hormone quantification or placed in liquid nitrogen and stored in a−80°C freezer for RNA extraction and gene expression analysis. The dry matter analysis was performed using two methods: the coring method and the F‐751 Avocado Quality Meter. Approximately 5 g from each side of the fruit's equator was removed using a 13.2‐mm coring cylinder and placed in a drying oven at 60°C for 36 h. Fruit samples were dried until a constant weight was achieved, after which the dry matter content was calculated using the standard procedure (Ranney et al. 1992). The results were recorded as a percentage of the original weight and reported as an average of the four individual values.

The F‐751 (Felix Instruments, Camas, USA) Avocado Quality Meter, which is equipped with a visible and near‐infrared (Vis‐NIR) spectrometer that operates in the 640–1050 nm range, captures dry matter data without destroying the fruit.

### 2.3 Nucleic Acid Extraction and qRT‐PCR Assays

Total RNA extraction, DNase treatment, and cDNA synthesis were performed as described previously (El Kayal et al. 2017). Quantitative reverse transcription polymerase chain reaction (qRT‐PCR) was conducted for 16 genes chosen to represent a variety of biological functions during the ripening and hormone biosynthesis. Gene‐specific primers were designed using Primer Express (v3.0, Applied Biosystems, Carlsbad, CA) (Supporting Information Table S2). Two micrograms of total RNA were treated with DNaseI (Invitrogen, Burlington, ON) prior to cDNA synthesis using Superscript II reverse transcriptase (Invitrogen). Polymerase chain reactions were performed in 10 μL containing SYBR Green master mix [0.2 mmol L^−1^ dNTPs, 0.3 U Platinum Taq Polymerase (Invitrogen), 0.25× SYBR Green, and 0.1× ROX], 20 ng of cDNA, and 300 nmol L^−1^ of each primer. Three biological and three technical replicates for each reaction were analyzed on a CFX96 Real‐Time PCR Detection System (BioRad, Mississauga, ON) with a first step of 95°C for 2 min followed by 40 cycles of 95°C for 15 s and 60°C for 1 min. Melting curves were generated using the following program: 95°C for 15 s, 60°C for 15 s, and 95°C for 15 s. Specific primers for β‐actin (FvAct) and GAPDH were used as the internal controls for the normalization of the RNA steady‐state level, and the relative changes in gene expression were quantified using the 2 − ΔΔCt method (Livak and Schmittgen 2001).

### 2.4 Detection and Quantitation of Phytohormones Using UPLC/MS

Samples (300 mg) were then suspended in 0.5 mL of extraction solvent consisting of 75% methanol (MS grade, Fisher Scientific, Canada; MeOH) and 5% formic acid (MS grade, Fisher Scientific, Canada) in Milli‐Q water. Samples were then held at −20°C for 1 h, centrifuged (15 min, 4°C, 14,000 rpm) and the supernatant removed. A second extraction was performed on the same sample using similar conditions as described above, and the supernatants were pooled. The pooled supernatant was then evaporated to dryness using nitrogen gas in a fume cupboard. The dried samples were reconstituted with 200 μL of buffer solution (formic acid: acetonitrile = 95:5) and then filtered through a 0.22 μM centrifuge filter (Millipore; 1 min, 13,000 rpm). The filtered supernatant was then transferred to a 96‐well collection plate and centrifuged (15 min, 4°C, 14000 rpm), and 100 μL of supernatant was transferred to a polypropylene vial (300 μL, 12 × 32 mm). All standards were analytical grade and were purchased from Sigma Aldrich, Canada. Phytohormones were separated by reverse‐phase liquid chromatography (ultra‐performance liquid chromatography system (UPLC); LC‐40D MS, Shimadzu, Japan) by injecting a 5 μL aliquot of sample onto a Shim‐pack Scepter LC column (2.1 × 50 mm, 1.9 μm; Mandel Scientific Company, Guelph, ON, Canada). Metabolites were separated with a gradient of solvents A (0.1% formic acid) and B (100% methanol), with initial conditions of 95% A (5% B) increasing to 5% A (95% B) over 4 min using a curve of 0. The column temperature was 40°C, and the flow rate was 0.2 mL/min. Metabolite peaks were identified by comparison with standards and quantified from a standard curve generated using a similar separation method and gradient conditions. Phytohormones were detected using a single quadrupole mass spectrometer (LCMS 2020, Shimadzu, Japan) in single ion recording (SIR) mode. Phytohormones such as SA (137 m/z) and GA3 (345 m/z) were detected in negative mode, whereas zeatin (220 m/z) and ABA (265 m/z) were detected in positive mode. In all cases, the probe temperature was set at 250°C with a gain of 5; the capillary voltage (positive and negative) was set at 0.5 kV. The instrument limits of detection were 1.52, 1.52, 6.1, and 0.925 for SA, zeatin, GA3, and ABA respectively, and the method limits of detection were 5.06 pg/g, 5.06 pg/g, 20.34 pg/g, and 1.27 pg/g. The linear range for each analyte was 5.94 pg/mL–6.25 μg/mL, 5.94 pg/mL–6.25 μg/mL, 24.4 pg/mL–25 μg/mL, 1.52 pg/mL, and 1.56 μg/mL for SA, ABA, GA3, and zeatin respectively.

### 2.5 Statistical Analysis

Data was collected for the analysis of fruit quality parameters and was analyzed using R Studio software (R.4.3.0); statistical analysis was conducted to compare phytohormones between varieties and assess parameters between the various locations in each variety. Based on the data obtained, a suitable distribution function was fit for the analysis. Error assumptions of the variance analysis (random, homogenous, normal distribution of error) were tested using the studentized residual plots and the Shapiro–Wilk normality test. Means for the analyses was determined using the LSMEANS statement, and means separation was conducted using the Tukey–Kramer adjusted multiple means comparison test. Outliers were analyzed using the studentized residuals to check for deviation by more than ±3.4. The type 1 error rate was set at 0.05 and 0.01 for all statistical comparisons. Furthermore, a regression analysis is conducted to ascertain the correlation between the dry matter content of avocado fruits as determined by the traditional approach and the non‐destructive NIR method. Lastly, principal component analysis graphs illustrate the maturity index correlation and clustering behavior with regard to varieties and locations. For parametric analysis, one‐way ANOVA followed by Tukey's Honestly Significant Difference (Tukey HSD) test was employed. This technique made it possible to determine that the phytohormones varied significantly between the varieties and between the locations in each variety. Also, to evaluate the significant difference of genes in each variety in the different locations and the significant difference of the post‐harvest maturity indices between varieties and between locations in each variety. Additionally, the Kruskal Wallis test, a non‐parametric substitute for ANOVA, was run to take into consideration any possible violation of parametric assumptions. Then, post hoc Dunn's test was applied to assess pairwise differences across groups. *p*‐values of less than 0.05 were used to determine the statistical significance of differences. The thorough comparison was made possible by these meticulous statistical approaches.

## 3 Results and Discussion

### 3.1 Hormone Quantification

Lambhass, Pinkerton, Reed, Hass, and Fuerte avocado varieties were evaluated for their content of five hormones: Abscisic acid, Jasmonic acid, Salicylic acid, Gibberellic acid, and Zeatin, in six different locations in Lebanon. Table 1 represents the average values of all locations by variety. There was no significant difference in Abscisic acid (ABA) content between the studied varieties. The highest content was found in the Reed variety (41.2 ng/g DW), and the lowest content was found in the Hass variety (29.13 ng/g DW). This finding is consistent with previous research indicating that ABA levels can vary within species but tend to stabilize under similar environmental conditions (Davies et al. 2010).

**TABLE 1:** Abscisic Acid (ABA), Jasmonic acid (JA), Salicylic acid (SA), Gibberellic acid (GA3) and Zeatin concentrations (ng/g DW) in Avocado Lambhass, Pinkerton, Reed, Hass, and Fuerte varieties harvested from different locations in Lebanon.

Variety	ABA	JA	SA	GA3	Zeatin
Lambhass	39.69 ± 1.05	4.35 ± 1.23 ab	7.18 ± 1.95 b	3.21 ± 0.3	3.45 ± 0.45 a
Pinkerton	30.59 ± 6.26	2.78 ± 0.36 b	10.99 ± 2.91 ab	3.48 ± 0.88	4.1 ± 0.42 a
Reed	41.2 ± 4.61	7.1 ± 1.73 a	15.06 ± 2.88 ab	1.84 ± 0.27	1.84 ± 0.32 b
Hass	29.13 ± 2.58	3.93 ± 0.79 ab	23.23 ± 6.01 a	3.65 ± 0.29	2.86 ± 0.34 ab
Fuerte	36.99 ± 7.35	2.8 ± 0.93 b	9.52 ± 2.2 ab	2.33 ± 0.6	1.75 ± 0.31 b

*Note:* While comparing phytohormones in different varieties, means with the same letter are not significantly different from each other (*p*‐value > 0.05).

As for Jasmonic acid (JA), the Reed variety had significantly the highest content (7.1 ng/g DW) compared to Pinkerton and Fuerte, which had the lowest JA contents with 2.78 ng/g DW and 2.8 ng/g DW, respectively. This suggests that Reed may have enhanced pest resistance, aligning with studies showing that JA levels correlate with defense responses (Creelman and Mullet 1997).

The Hass variety recorded the significantly highest Salicylic acid (SA) values (23.23 ng/g DW), whereas Lambhass recorded the significantly lowest SA values (7.18 ng/g DW). There is very little data available regarding the variability in hormone content among avocado varieties, but this study suggests that Hass may be better equipped to resist pathogens, supporting findings that SA is integral to plant immune responses (Davies et al. 2010). Zeatin, a type of cytokinin, promotes cell division and growth. The Pinkerton, Lambhass, and Hass varieties had significantly higher Zeatin contents compared to Reed and Fuerte, suggesting that the former varieties may exhibit more vigorous growth. It is important to mention that the differences in hormone content among avocado varieties can be attributed to a combination of genetic factors and agricultural practices. Soil management, irrigation, pest control, and harvest timing all play crucial roles in determining the biochemical composition of the fruits. By optimizing these practices, farmers can influence hormone levels, ultimately improving fruit quality and resilience. To include the effect of different locations, hormone quantification was performed on all avocado varieties from their respective growing areas. Table 1 presents the average hormone content in avocado varieties across various locations. Detailed hormone content for each variety per location can be found in Supporting information Table S3.

The hormone content in avocado varieties across different locations in Lebanon reveals significant variability influenced by agricultural practices, environmental conditions, and altitude. Abscisic acid (ABA) levels are highest in Aadbel (600 m) and Beit Mallat (540 m), indicating these higher altitude areas may experience more environmental stressors such as water scarcity and poorer soil quality. ABA is a stress hormone, and its elevated levels are indicative of plants' response to adverse conditions (Davies et al. 2010). Jasmonic acid (JA) content is significantly higher in Abbasiyeh (158 m) and Ansar (302 m), suggesting increased pest pressures at these lower altitudes, which require enhanced plant defense mechanisms (Creelman and Mullet 1997).

Salicylic acid (SA) levels are notably elevated in Aadbel and Qloud El Barka (332 m), which could be due to a robust pathogen defense response in these regions, possibly influenced by the local environmental conditions and altitude. In contrast, Beit Mallat and Abbasiyeh exhibit lower SA levels, indicating less pathogen pressure or different pest management practices in these areas. Gibberellic acid (GA3) levels are relatively consistent across most locations, with the highest levels in Nmeiriyeh (320 m) and the lowest in Qloud El Barka, suggesting stable growth conditions but potential growth limitations at higher altitudes. Lower GA3 levels in Qloud El Barka might suggest environmental stressors or different agricultural practices affecting growth.

Zeatin content, reflecting cell division and growth, is highest in Abbasiyeh and Ansar, indicating vigorous growth due to possibly favorable soil fertility and effective nutrient management in these areas (Zhao et al. 2005). The variations in hormone content across these locations underscore the importance of optimized agricultural practices tailored to specific environmental conditions. For instance, areas with higher ABA levels might benefit from improved irrigation practices to reduce water stress, while regions with elevated JA and SA levels could focus on enhanced pest and disease management strategies. Furthermore, the influence of altitude on hormone levels suggests that microclimatic conditions and altitude‐specific practices play a crucial role in determining the biochemical composition of avocados. Overall, tailored agricultural practices, including optimized irrigation, pest management, and soil fertility enhancements, are essential to improve avocado fruit quality and resilience across diverse growing conditions and altitudes (Salameh et al. 2022) (Table 2).

**TABLE 2:** This table represents the average hormone content in avocado varieties across different locations.

Location	ABA	JA	SA	GA3	Zeatin
Aadbel	63.3 ± 14.4 a	1.8 ± 0.3 b	27.9 ± 18.6	2 ± 0.6 b	2.08 ± 0.5
Abbasiyeh	28.4 ± 4.1 cd	5.7 ± 1.5 a	9.9 ± 2.7	2.04 ± 0.3 b	3.9 ± 0.6
Ansar	21.2 ± 2.2 c	6.4 ± 0.9 a	9.9 ± 0.6	3.6 ± 0.7 b	3.3 ± 0.8
Beit Mallat	43.9 ± 1.5 ab	2.5 ± 0.9 ab	6.6 ± 1	4.1 ± 0.8 b	2.3 ± 0.9
Mrwaniyeh	32.5 ± 4.1 d	2.4 ± 0.3 ab	14.9 ± 6.6	3.4 ± 0.3 b	2.3 ± 0.7
Nmeiriyeh	30.3 ± 11.3 bcd	3.01 ± 1.4 ab	11.9 ± 1.8	5.1 ± 2.2 b	3.4 ± 1.5
Qloud El Barka	26.4 ± 0.7 cd	1.7 ± 0.8 b	16.06 ± 15.1	0.7 ± 0.06 a	2.2 ± 1.2

*Note:* The values are given as mean ± standard deviation, and different letters indicate significant differences between the locations for each hormone.

### 3.2 Gene Expression

With the aim of identifying and highlighting the main hormonal and molecular events governing the process of avocado fruit ripening, qRT‐PCR was conducted for a set of 16 genes. The expression levels of these genes were quantified in the five chosen varieties growing at varying altitudes in different locations, elucidating the hormonal and molecular mechanisms underlying this process.

**FIGURE 2:**
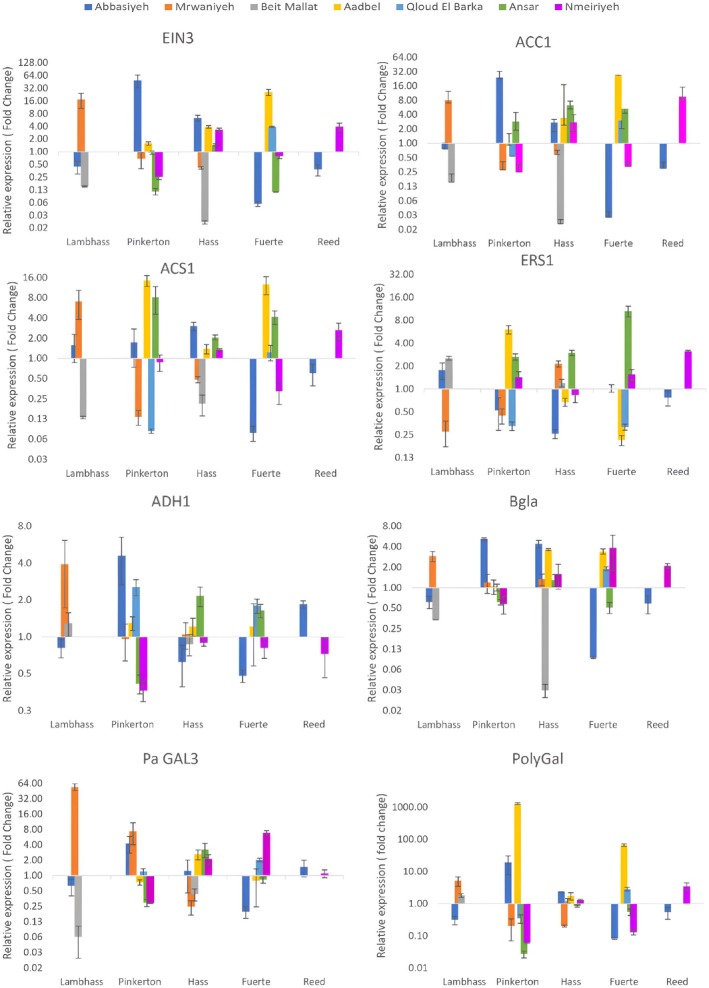
Expression profiles of PaERS1, PaEIN3, PaGTR, PaCTR1, PaADH1, PaADH2, PaADH3, and PaG3PD genes of Lambhass, Pinkerton, Hass, Fuerte, and Reed avocado varieties harvested from seven different locations in Lebanon: Aadbel, Abbasiyeh, Ansar, Nmeiriyeh, Mrwaniyeh, Beit Mallat, and Qloud El Barqa. Relative gene expression was calculated using 2^−∆∆CT^ method (fold change).

**FIGURE 3:**
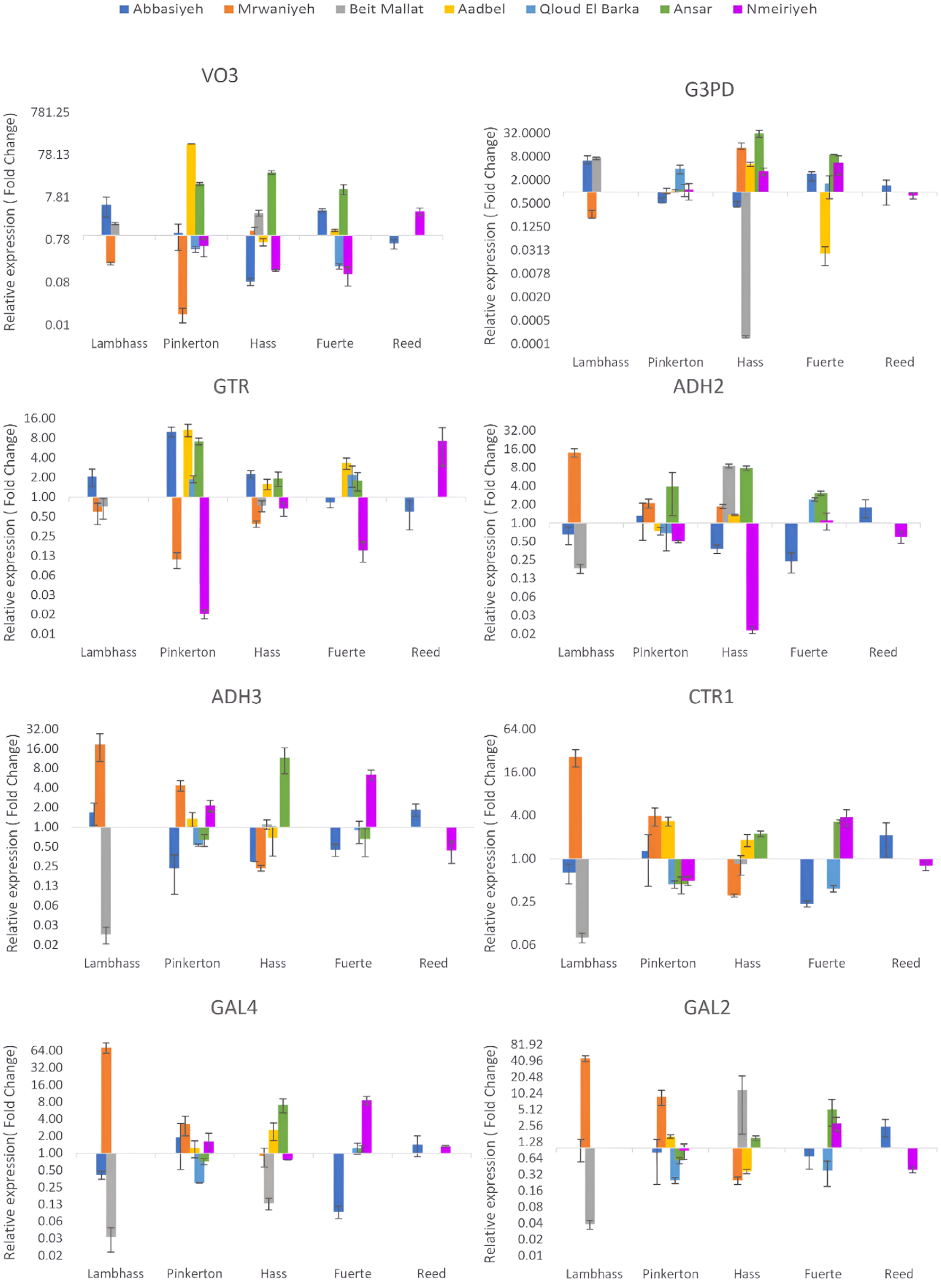
Expression profiles of PaGAL2, PaGAL3, PaGAL4, PaACS1, PaACC1, PaVO3, PaBGLA, and PaPolyGal genes of Lambhass, Pinkerton, Hass, Fuerte, and Reed avocado varieties harvested from seven different locations in Lebanon: Aadbel, Abbasiyeh, Ansar, Nmeiriyeh, Mrwaniyeh, Beit Mallat, and Qloud El Barqa. Relative gene expression was calculated using 2^−∆∆CT^ method (fold change).

Notably, *PaEIN3* expression was exceptionally high in Abbasiyeh for Pinkerton and Hass varieties, with fold changes of 49.48 and 16.50, respectively, highlighting a strong regulatory role under these conditions (Figure 2). Conversely, low expression levels were seen in Fuerte and Reed in Abbasiyeh and Mrwaniyeh, indicating location‐specific transcriptional regulation (Figure 2). *PaACC1* exhibited the highest expression in Aadbel for Fuerte, suggesting a significant role in ethylene biosynthesis at this location (Figure 3). This contrasted with the low expression observed in Reed across most locations, except Nmeiriyeh, underscoring varietal differences in ethylene production pathways. Similarly, *PaACS1* was highly expressed in Ansar for Pinkerton and Hass Fuerte, emphasizing its pivotal role in ethylene synthesis in these varieties, while the lowest expression was recorded in Beit Mallat, indicating less ethylene synthesis activity there. *PaERS1* transcript level peaked in Ansar for Fuerte and Hass, correlating with an enhanced ethylene response in these varieties at this location. The lowest levels were observed in Aadbel for Fuerte, suggesting a reduced ethylene response. *PaADH1* was most highly expressed in Abbasiyeh for Pinkerton, pointing to a significant role in ethanol metabolism at this location, while lower expression levels in Hass and Fuerte in Abbasiyeh indicated variation in metabolic pathways. *PaBgla* expression was highest in Abbasiyeh for Pinkerton and Hass, implying a role in cell wall modification and fruit softening, with the lowest levels observed in Fuerte and Reed in Abbasiyeh. PaGAL3 exhibited the highest expression in Mrwaniyeh for Lambhass, indicating an essential role in galactosidase activity, while the lowest expression in Hass in Mrwaniyeh suggested different regulatory mechanisms. Finally, *PaPolyGal* showed the highest expression in Aadbel for Pinkerton and Fuerte, likely reflecting its role in cell wall degradation and fruit ripening, with low levels in Abbasiyeh for Lambhass and Reed, indicating a lesser role in these processes.

As shown in Figure 3, for instance, *PaVO3* expression was notably high in Aadbel for the Pinkerton variety, with a fold change of approximately 781.25, indicating a strong regulatory role in this location. Conversely, low *PaVO3* expression levels were observed in Fuerte and Reed across multiple locations, suggesting location‐specific transcriptional regulation.

Similarly, *PaG3PD* showed the highest expression in Abbasiyeh for Lambhass, suggesting a critical role in glycerol‐3‐phosphate dehydrogenase activity in this variety at this location. The lowest G3PD expression levels were seen in Hass, Fuerte, and Reed, highlighting varietal differences in metabolic pathways involving glycerol‐3‐phosphate. *PaGTR* expression was lowest in Mrwaniyeh for Pinkerton and Lambhass, indicating a moderate role in glucosinolate transport in these varieties under these conditions, whereas the lowest expression was noted in Nmeiriyeh for Pinkerton and Fuerte, reflecting less activity of this gene in these conditions.

*PaADH2* and *PaADH3* exhibited notable expression patterns, with *PaADH2* showing the highest expression in Mrwaniyeh for Lambhass, suggesting significant ethanol metabolism activity in this variety at this location. Conversely, *PaADH3* expression peaked in Mrwaniyeh for Lambhass and Pinkerton, indicating variation in alcohol dehydrogenase isoform activity across different locations and varieties. Lower expression levels of both *PaADH2* and *PaADH3* in other varieties and locations reflect differences in ethanol metabolism pathways. *PaCTR1* expression was highest in Mrwaniyeh for Lambhass, highlighting a strong regulatory role in the ethylene signaling pathway in this variety at this location. Low *PaCTR1* expression levels in Reed across most locations underscore varietal differences in ethylene signaling. Similarly, the expression of *PaGAL2* and *PaGAL4* was notably high in Mrwaniyeh for Lambhass, suggesting an essential role in galactosidase activity at this location. The lowest expression levels for both genes were observed in other varieties and locations, indicating differential regulation of galactosidase activity.

These findings suggest that the transcriptional regulation of these genes is highly influenced by both the variety of avocado and the environmental conditions of the growing location, necessitating further research to elucidate the underlying mechanisms and their implications for avocado cultivation and post‐harvest quality. The data suggest that gene expression related to fruit ripening in avocados is highly dependent on both the variety and the geographical location. Genes such as *PaEIN3* and *PaACC1*, involved in ethylene signaling, show significant location‐specific expression, implying environmental factors like altitude and climate could influence ethylene production and response. This aligns with previous studies indicating that ethylene biosynthesis and response genes are tightly regulated by environmental conditions (Zhu et al. 2011; Barry and Giovannoni 2007). The high expression of *PaACS1* and *PaERS1* in specific locations further underscores the role of ethylene in ripening, with varying levels suggesting differential regulation of ethylene synthesis and signaling pathways across different environments. This is consistent with the findings of McMurchie et al. (1972), who demonstrated the critical role of ethylene in fruit ripening and its regulation by environmental factors. Moreover, the variable expression of *PaADH1* and *PaBgla* highlights the complexity of metabolic pathways during ripening, which are influenced by both intrinsic genetic factors and extrinsic environmental conditions. The role of alcohol dehydrogenase in fruit aroma and flavor development has been well documented (Defilippi et al. 2005), and the observed variations support the notion that metabolic pathways are differentially regulated in response to environmental stimuli. Lastly, the significant expression of PolyGal in Aadbel suggests robust cell wall modification processes in this location, critical for fruit softening and ripening (Brummell and Harpster 2001). This differential expression could be crucial for post‐harvest handling and storage strategies tailored to specific growing conditions.

The high variability in gene expression, particularly for genes involved in ethylene biosynthesis (*PaACC1, PaACS1*), ethylene signaling (*PaERS1, PaCTR1*), and cell wall modification (*PaBgla, PaPolyGal*), points to the complex regulation of ripening processes. For instance, *PaEIN3*, a key regulator of ethylene response, showed significant variation across locations and varieties, indicating its role in fine‐tuning the ripening process in response to environmental cues. Similarly, the differential expression of *PaADH* isoforms (ADH1, ADH2, ADH3) across varieties and locations highlights the complexity of ethanol metabolism during ripening. The significant expression of galactosidases (*PaGAL3, PaGAL2, PaGAL4*) in specific varieties and locations further emphasizes the role of these enzymes in cell wall degradation and fruit softening, crucial for ripening.

These findings align with previous studies demonstrating the impact of environmental factors on gene expression and fruit quality in avocados. For example, studies have shown that ethylene production and response can vary significantly with environmental conditions, affecting fruit ripening and post‐harvest quality (Yahia 2009; Hershkovitz et al. 2005). The observed variability in gene expression underscores the need for further research to elucidate the underlying mechanisms and their implications for avocado cultivation and post‐harvest management.

### 3.3 Physicochemical Characteristics

Physicochemical maturity indices were measured in the five varieties growing in the different locations. In Table 4, the average of each maturity index is calculated as a mean from all the studied locations. The Reed variety showed the highest weight value, reaching 299.6 g, while Hass was the lightest, noting 163.48 g. Similarly, Reed and Hass also exhibited the highest and least values in diameter, reaching 7.7 cm and 5.85 cm, respectively. In contrast, the length of Pinkerton avocado exhibited the highest measurements, 13.37 cm, while Reed marked the shortest length of 8.85 cm, but this is due to the nature of the rounded variety. Dry matter is one of the most important maturity indices in avocado as it determines the ripening status and thus the harvesting time. In this study, Hass and Fuerte have the highest dry matter of about 25% while noting the least values in Reed, reaching 20.43% (Table 3).

**TABLE 3:** Physicochemical characteristics including (weight (g), diameter (cm), length (cm), dry matter (%), firmness (KgF), and caliber (mm)) of five avocado varieties (Lambhass, Pinkrton, Reed, Hass, and Fuerte) grown in south and north Lebanon.

Variety	Fruit weight	Diameter	Length	Dry matter	Firmness	Caliber
Lambhass	194.65 ± 3.72 d	6.18 ± 0.06 c	9.1 ± 0.07 d	24.14 ± 0.43 b	3.84 ± 0.07 c	65.32 ± 0.78 bc
Pinkerton	239.51 ± 3.31 b	6.31 ± 0.04 b	13.37 ± 0.09 a	23.01 ± 0.22 b	4.14 ± 0.05 ab	65.93 ± 1.52 bc
Reed	299.6 ± 4.9 a	7.7 ± 0.05 a	8.85 ± 0.06 d	20.43 ± 0.49 c	3.54 ± 0.05 d	80.6 ± 0.82 a
Hass	163.48 ± 1.89 d	5.85 ± 0.03 d	9.43 ± 0.05 c	25.84 ± 0.22 a	4.35 ± 0.06 a	62.07 ± 0.52 c
Fuerte	227 ± 2.51 c	6.46 ± 0.04 bc	11.22 ± 0.06 b	25.3 ± 0.19 a	3.76 ± 0.1 bc	66.8 ± 0.69 b

*Note:* Values followed by the same letter are not significantly different at *p* ≥ 0.05 level. Variables were considered significant at < 0.05 risk level.

Given the importance effect of the altitudes, geographical locations and agricultural practices on the physicochemical characteristics, data analysis was performed in all varieties per location as shown in Table 4. Some prominent trends were observed for some of the studied varieties. For instance, Fuerte growing in the northern location “Aadbel” was shown that the highest values of fruit weight, diameter, length, and dry matter reaching 281.6 g, 7.4 cm, 12.52 cm, 30.83%, respectively (Table 4). It is important to mention that Aadbel has the longest ripening season which might explain why Fuerte in this location reached the lowest firmness value of 2.74 KgF, significantly different from the southern location “Mrwaniyeh” which exhibited the firmest Fuerte fruits with 5.46 KgF. Another interesting pattern, in the northern location “Beit Mallat” recorded the highest dry matter percentage in Hass, reaching 29.97%, significantly different to all southern locations Abbasiyeh, Nmeiriyeh, and Mrwaniyeh ranging from 23% to 25%. This finding is consistent with other studies when observed that fruits grown at higher altitudes often have higher dry matter content due to cooler temperatures and higher light intensities, which enhance photosynthesis and carbohydrate accumulation. For instance, avocados grown at higher altitudes in Mexico have been reported to exhibit higher dry matter content, which is associated with better taste and nutritional quality (Bertling and Bower 2006). Other studies on avocados as well have shown that geographical location impacts oil content and fatty acid composition. Avocados grown in regions with higher altitudes and cooler climates typically have higher oil content and better flavor profiles. This is due to the slower maturation process, allowing for greater accumulation of oil and beneficial fatty acids (Whiley et al. 2002).

**TABLE 4:** Post‐harvest maturity indices (weight (g), diameter(cm), length(cm), dry matter (%), firmness (KgF)s, and caliber(mm)) of 5 avocado varieties (Lambhass, Pinkrton, Reed, Hass, and Fuerte) compared among 6 locations in south and north Lebanon (Abbasiyeh, Ansar, Nmeiriyeh, Qloud El Brka, Mrwaniyeh, and Aaadbel), between October 2021 and April 2022.

Variety	Location	Fruit weight	Diameter	Length	Dry matter	Firmness	Caliber
Fuerte	Aadbel	281.6 ± 20.14 a	7.4 ± 0.42 a	12.52 ± 0.7 a	30.83 ± 0.93 a	2.74 ± 0.16 b	67.87 ± 3.01
	Abbasiyeh	234.12 ± 7.93 a	6.4 ± 0.09 b	10.89 ± 0.18 b	23.77 ± 0.65 b	3.48 ± 0.12 b	68.88 ± 2.94
	Ansar	214.39 ± 3.54 b	6.21 ± 0.05 b	11.11 ± 0.1 b	24.6 ± 0.27 b	3.5 ± 0.06 b	67.92 ± 1.95
	Nmeiriyeh	238.09 ± 4.92 a	6.48 ± 0.07 b	11.38 ± 0.11 b	25.74 ± 0.41 b	3.99 ± 0.08 b	65.97 ± 0.62
	Mrwaniyeh	207.78 ± 4.3 b	6.08 ± 0.06 b	10.99 ± 0.13 b	25.35 ± 0.33 b	5.46 ± 0.26 a	64.43 ± 0.45
	Beit Mallat	186 ± 16.8 b	6.2 ± 0.19 b	10.44 ± 0.37 b	24.53 ± 0.77 b	3.36 ± 0.1 b	66.87 ± 0.62
Hass	Aadbel	158.4 ± 8.62 bc	5.86 ± 0.13	10.06 ± 0.25 a	26.35 ± 1.52 ab	4.65 ± 0.21 a	62.97 ± 0.42
	Abbasiyeh	165.27 ± 2.78 b	5.79 ± 0.04	9.38 ± 0.12 b	23.85 ± 0.46 c	4.54 ± 0.09 ab	63.88 ± 1.51 ab
	Ansar	141.42 ± 3.1 c	5.53 ± 0.06	9.12 ± 0.1 b	25.34 ± 0.38 ab	4.26 ± 0.1 ab	60.43 ± 1.61 b
	Nmeiriyeh	193.31 ± 3.55 a	6.15 ± 0.06	9.97 ± 0.09 a	24.72 ± 0.44 b	4.63 ± 0.14 a	64.23 ± 0.56 a
	Mrwaniyeh	175.51 ± 3.34 b	5.93 ± 0.05	9.67 ± 0.1 b	24.81 ± 0.5 b	4.63 ± 0.11 a	62.4 ± 0.45 ab
	Beit Mallat	147 ± 11.54 bc	5.92 ± 0.08	8.38 ± 0.41 c	29.97 ± 0.18 a	3.38 ± 0.21 b	59.4 ± 1.6 b
Lambhass	Abbasiyeh	144.97 ± 2.38 b	5.7 ± 0.05 a	8.3 ± 0.08 b	22.05 ± 0.57	3.95 ± 0.1	61 ± 1.26 b
	Mrwaniyeh	211.58 ± 5.08 a	6.65 ± 0.07 b	9.1 ± 0.1 a	22.6 ± 0.66	4.1 ± 0.11	66.97 ± 0.88 a
	Beit Mallat	227.4 ± 5.43 a	6.5 ± 0.08 b	9.9 ± 0.17 a	27.77 ± 0.2	3.47 ± 0.13	68 ± 0.71 a
Pinkerton	Abbasiyeh	196.77 ± 4.06 d	5.84 ± 0.05 c	11.68 ± 0.14 c	21.8 ± 0.4 b	4.56 ± 0.09 a	67.16 ± 2.71 b
	Ansar	241.05 ± 5.3 b	6.25 ± 0.06 b	13.53 ± 0.15 a	22.45 ± 0.41 b	3.89 ± 0.07 c	67.74 ± 2.24 b
	Nmeiriyeh	280.54 ± 6.25 a	6.77 ± 0.06 a	13.89 ± 0.17 a	25.29 ± 0.38 a	4.43 ± 0.07 ab	69.07 ± 2.39 a
	Qloud El Barka	232.2 ± 7.64 c	6.32 ± 0.07 ab	13.4 ± 0.55 ab	20.27 ± 0.86 b	3.56 ± 0.15 bc	67.07 ± 2.39 b
	Mrwaniyeh	254.49 ± 7.27 b	6.52 ± 0.07 ab	12.78 ± 0.14 b	22.91 ± 0.42 b	4.51 ± 0.12 a	59.76 ± 4.29 c
	Aadbel	232 ± 27.97 c	6.16 ± 0.33 abc	14.94 ± 0.42 a	25.35 ± 0.84 a	3.87 ± 0.6 abc	68.07 ± 2.39 b
Reed	Abbasiyeh	288.72 ± 6.43 b	7.48 ± 0.07	8.75 ± 0.08	20.98 ± 0.75	3.5 ± 0.06	80.82 ± 1.37
	Nmeiriyeh	310.48 ± 7.17 a	7.92 ± 0.07	8.94 ± 0.08	19.88 ± 0.62	3.58 ± 0.08	80.38 ± 0.99

*Note:* Values followed by the same letter are not significantly different at *p* ≥ 0.05 level. Variables were considered significant at < 0.05 risk level.

Our previous study Salameh et al. (2022) shed light on the impact of the geographical location on the physicochemical characteristics. Temperature variations associated with different altitudes significantly affect the ripening process and quality of climacteric fruits. Higher altitudes generally experience cooler temperatures, which can slow down the ripening process, leading to an extended growing period. This extended period often results in fruits with better flavor, higher sugar content, and improved overall quality due to the prolonged synthesis and accumulation of secondary metabolites (Brizzolara et al. 2020). Finally, fruit caliber, which refers to the size and diameter of the fruit, is slightly influenced by altitude and geographical location in a similar manner to fruit weight. Higher altitudes tended to produce fruits with smaller calibers due to slower growth rates, while lower altitudes favor larger calibers due to more rapid growth. This has been observed in fruits such as apples and tomatoes, where those grown at higher altitudes are often smaller but have higher quality attributes like firmness and flavor concentration.

**FIGURE 4:**
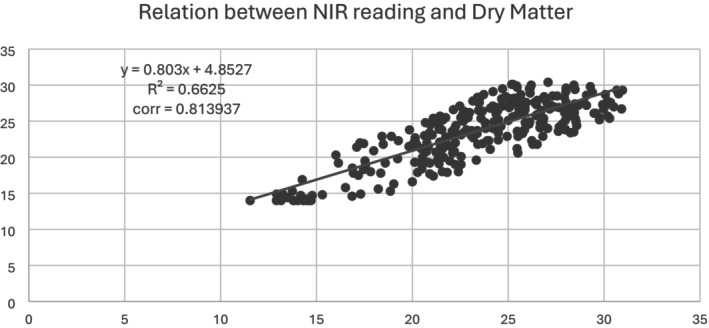
Correlation between the dry matter of avocado fruits measured by the conventional method (oven) and the NIR non‐destructive method.

In the context of fruit quality, dry matter content is a critical parameter, influencing attributes such as texture, taste, and shelf‐life. Dry matter content typically correlates with improved fruit firmness and better overall eating quality (Harker et al. 2002). The scatter plot illustrates in Figure 4 a strong positive relationship between Near‐Infrared (NIR) readings and dry matter content in avocado fruit samples, as evidenced by the linear regression equation 𝑦 = 0.803𝑥 + 4.8527. The coefficient of determination (𝑅^2^ = 0.6625) indicates that 66.25% of the variability in dry matter content is explained by NIR readings, while the correlation coefficient of 0.813937 underscores a strong positive linear relationship. This significant correlation suggests that NIR spectroscopy can be a reliable non‐destructive method for predicting dry matter content, an essential indicator of fruit quality and ripeness. The application of NIR spectroscopy in determining dry matter content is supported by various studies. Bellon‐Maurel and McBratney (2011) highlighted the advantages of NIR spectroscopy in agricultural applications, including its rapidity, accuracy, and non‐destructive nature, making it suitable for real‐time monitoring and quality control. Similarly, Pandiselvam et al. (2022) demonstrated the efficacy of NIR spectroscopy in assessing the quality attributes of various fruits, emphasizing its potential in enhancing post‐harvest handling and processing. Therefore, the ability to accurately predict dry matter content using NIR spectroscopy provides significant advantages in the fruit industry, enabling producers to ensure consistent quality and optimize harvest timing. Furthermore, the use of NIR spectroscopy aligns with sustainable agricultural practices by reducing waste and improving efficiency. Traditional methods of assessing dry matter content often involve destructive sampling, which can be both time‐consuming and resource‐intensive. In contrast, NIR spectroscopy offers a swift and non‐invasive alternative, facilitating large‐scale assessments without compromising the integrity of the fruit.

In summary, the strong correlation between NIR readings and dry matter content demonstrated in the scatter plot underscores the potential of NIR spectroscopy as a valuable tool in fruit quality assessment. Our results highlight the potential benefits of near‐infrared (NIR) sensors in agricultural contexts, providing accurate and non‐destructive ways of evaluating avocado fruit development, reducing fruit waste, and enhancing harvesting techniques.

**FIGURE 5:**
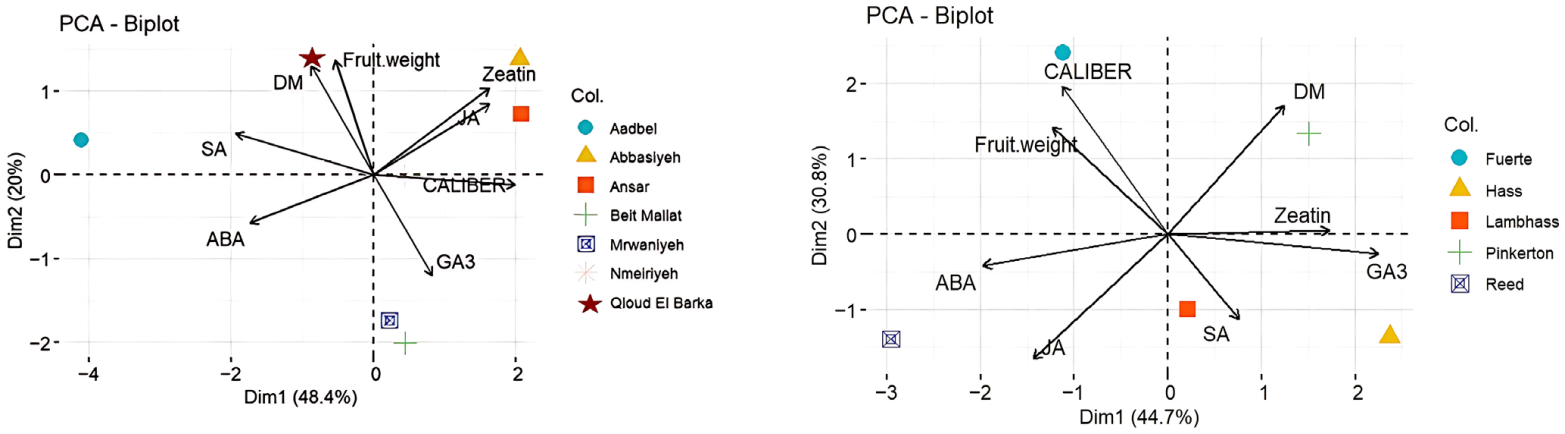
(A, B) Principal component analysis graphs showing the correlation and clustering behavior of the studied maturity indices with respect to varieties and locations.

The Principal Component Analysis (PCA) biplot (Figure 5A,B) provides a comprehensive visualization of the relationships between various fruit traits (caliber, dry matter content, and fruit weight) and hormonal contents (Zeatin, GA3, SA, ABA, and JA) across different fruit varieties (Fuerte, Hass, Lambhass, Pinkerton, and Reed). The two principal components, Dim1 and Dim2, together explain 75.5% of the variance in the dataset. Dim1 (44.7%) primarily distinguishes fruit varieties based on the levels of growth‐promoting hormones (GA3, Zeatin, and SA), while Dim2 (30.8%) separates the varieties based on traits like fruit caliber and dry matter content. Caliber and dry matter content are highly correlated with Dim2, indicating their importance in differentiating fruit varieties along this axis. Varieties with higher caliber and dry matter content are positioned positively on Dim2. Zeatin, GA3, and SA are strongly associated with Dim1, suggesting that higher levels of these hormones contribute significantly to the variation captured by this component. Conversely, ABA and JA are negatively correlated with Dim1, implying that varieties with higher levels of these hormones are distinct from those with elevated GA3 and Zeatin. Fruit weight shows a slight negative correlation with Dim1, indicating an inverse relationship with GA3 and Zeatin levels. Among the varieties, Fuerte, positioned positively on Dim2 and negatively on Dim1, is characterized by larger fruit caliber and higher dry matter content but lower levels of GA3 and Zeatin. Hass, located positively on Dim1, is associated with higher levels of GA3 and Zeatin, highlighting its growth‐promoting hormone content. Lambhass, found negatively on Dim1, shows higher levels of ABA and JA, emphasizing its unique hormonal profile. Pinkerton, positioned positively along both dimensions, balances high dry matter content with significant levels of hormones like Zeatin and GA3. Reed, situated negatively on Dim2 and slightly positively on Dim1, has lower dry matter content and fruit caliber but moderate associations with GA3 and Zeatin. The biplot reveals positive correlations among variables like Zeatin, GA3, and SA, and negative correlations between variables like ABA and GA3. This PCA biplot effectively illustrates the multidimensional relationships between fruit characteristics and hormonal contents across various fruit varieties, highlighting crucial insights for selecting and breeding fruit varieties with desirable traits under different environmental conditions. The biplot in Figure 5B illustrates the relationships between the various fruit traits (caliber, dry matter content, and fruit weight) and hormonal contents (Zeatin, GA3, SA, and ABA) across the geographical locations. The two principal components, Dim1 and Dim2, explain 68.4% of the variance in the dataset, with Dim1 accounting for 48.4% and Dim2 for 20%. Dim1 primarily differentiates locations based on the levels of growth‐promoting hormones (Zeatin and GA3) and fruit weight, while Dim2 separates them based on dry matter content (DM). Fruit weight and dry matter content are positively correlated with Dim2, indicating their importance in differentiating the locations along this axis. Locations with higher fruit weight and dry matter content are positioned positively on Dim2. Zeatin and GA3 are strongly associated with Dim1, suggesting that higher levels of these hormones contribute significantly to the variation captured by this component. Conversely, ABA and SA are negatively correlated with Dim1, implying that locations with higher levels of these hormones are distinct from those with elevated Zeatin and GA3 levels. Qloud El Barka, marked by a red star and positioned positively on both Dim1 and Dim2, is characterized by larger fruit weight, higher dry matter content, and significant levels of Zeatin and GA3, indicating favorable conditions for fruit growth and hormone production. Abbasiyeh, located positively on Dim1, is associated with high levels of Zeatin and GA3, highlighting its growth‐promoting hormone content. Aadbel, positioned negatively on Dim1, shows higher levels of ABA and SA, emphasizing its unique hormonal profile. Ansar, situated positively on Dim2, has higher dry matter content, indicating favorable conditions for fruit firmness. Beit Mallat, positioned negatively on Dim2 and slightly negatively on Dim1, has lower dry matter content and fruit weight but moderate associations with SA and ABA.

The biplot reveals positive correlations among variables like Zeatin and GA3, and negative correlations between variables like ABA and GA3. This PCA biplot effectively illustrates the multidimensional relationships between fruit characteristics and hormonal contents across various geographical locations, highlighting crucial insights for selecting and breeding fruit varieties with desirable traits under different environmental conditions.

## 4 Conclusion

The main findings of this study revealed a significant correlation between the expression levels of certain genes and hormone levels, which varied depending on both the avocado variety and the location. Phytohormone levels showed more substantial variation across locations altitudes than among the six avocado varieties. Physicochemical characteristics were also assessed, and a principal component analysis indicated a positive correlation between certain phytohormones and maturity indices, influenced by both variety and location.

Overall, the study enhances our comprehension of how phytohormones, altitude, fruit maturity, and ripening processes interact across five widely cultivated avocado varieties. This understanding is crucial for optimizing avocado cultivation and improving fruit quality.

## Conflicts of Interest

The authors declare no conflicts of interest.

## Supporting information


**Figure S1:** Map of the south (Nmeiriyeh, Marwanieh, Ansar, and Abbasiyeh) and north locations (Aadbel, Beit Mallat and Qloud El Barka) used in this study and their altitudes.

**Table S1:** Variety names and locations used in this study along with and their GPS units.

**Table S2:** Gene Name and the Primers List Used in This Study Including the Accession Numbers and Gene Abbreviation.

**Table S3:** Abscisic Acid (ABA), Jasmonic acid (JA), Salicylic acid (SA), Gibberellic acid (GA3) and Zeatin concentrations (ng/g DW) in Avocado varieties harvested from Aadbel, Abbasiyeh, Ansar, Nmeiriyeh and Qloud El Barqa locations in Lebanon. While comparing phytohormones in different locations for the same variety, means with the same letter are not significantly different from each other (*p*‐value > 0.05).

## Data Availability

The original contributions presented in the study are included in the article and supporting information; further inquiries can be directed to the corresponding author.
