# Supplementary figures and images for: Hormonal Regulation of Avocado (Persea americana) Across Altitudinal Gradients
Source: Plant Environ Interact. 2025 Sep 8;6(5):e70083. doi: 10.1002/pei3.70083 (PMC12415870; doi:10.1002/pei3.70083)

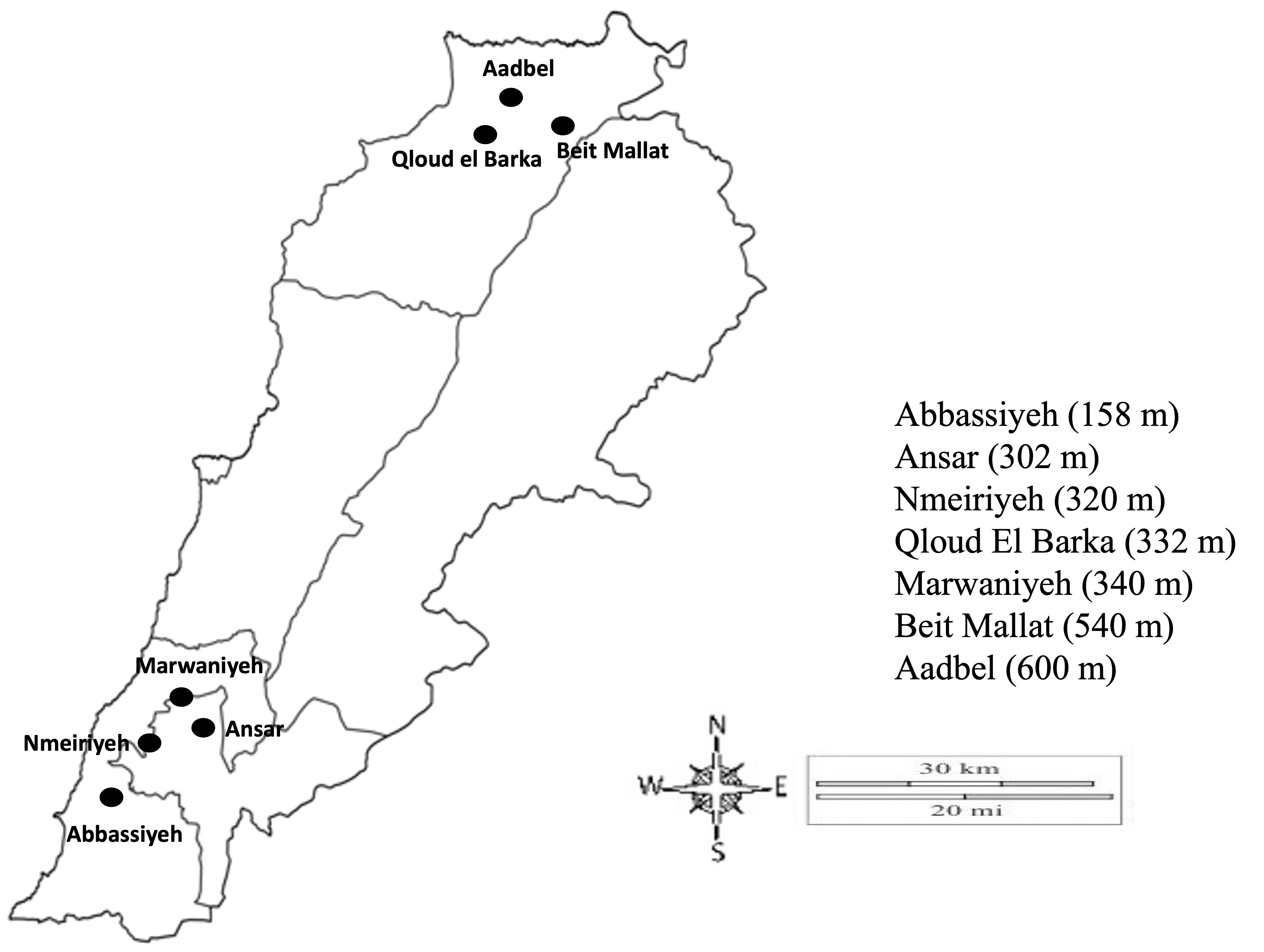

Supplement: Supplementary file 1 — Figure S1: Map of the south (Nmeiriyeh, Marwanieh, Ansar, and Abbasiyeh) and north locations (Aadbel, Beit Mallat and Qloud El Barka) used in this study and their altitudes. [file PEI3-6-e70083-s002.jpg]
