# Supplementary material for: Hormonal Regulation of Avocado (Persea americana) Across Altitudinal Gradients
Source: Plant Environ Interact. 2025 Sep 8;6(5):e70083. doi: 10.1002/pei3.70083 (PMC12415870; doi:10.1002/pei3.70083)
Supplement: Supplementary file 2 — Table S1: Variety names and locations used in this study along with and their GPS units. [file PEI3-6-e70083-s003.docx]

| **Variety** | **location** | **Longititude** | **Latitude** | **Altitude (m)** |
| --- | --- | --- | --- | --- |
| Fuerte | Aadbel | 34.5351819 | 36.095772 | 270 |
|  | Abbasiyeh | 33.300451 | 35.237454 | 50 |
|  | Ansar | 33.3956639 | 35.335851 | 299 |
|  | Nmeiriyeh | 33.418322 | 35.421759 | 400 |
|  | Qloud El Barka | 34.4966233 | 36.059505 | 333 |
| Hass | Aadbel | 34.5351819 | 36.095772 | 270 |
|  | Abbasiyeh | 33.300451 | 35.237454 | 50 |
|  | Ansar | 33.3956639 | 35.335851 | 299 |
|  | Beit Mallat | 34.529445 | 36.162893 | 582 |
|  | Mrwaniyeh | 33.4620556 | 35.357051 | 300 |
|  | Nmeiriyeh | 33.418322 | 35.421759 | 400 |
| Lambhass | Abbasiyeh | 33.300451 | 35.237454 | 50 |
|  | Beit Mallat | 34.529445 | 36.162893 | 582 |
|  | Mrwaniyeh | 33.4620556 | 35.357051 | 300 |
| Pinkerton | Aadbel | 34.5351819 | 36.095772 | 270 |
|  | Abbasiyeh | 33.300451 | 35.237454 | 50 |
|  | Ansar | 33.3956639 | 35.335851 | 299 |
|  | Mrwaniyeh | 33.4620556 | 35.357051 | 300 |
|  | Nmeiriyeh | 33.418322 | 35.421759 | 400 |
|  | Qloud El Barka | 34.4966233 | 36.059505 | 333 |
| Reed | Abbasiyeh | 33.300451 | 35.237454 | 50 |
|  | Nmeiriyeh | 33.418322 | 35.421759 | 400 |

Table S1: variety names and locations used in this study along with and their GPS units (Longitude and Latitude).
