# Supplementary material for: Hormonal Regulation of Avocado (Persea americana) Across Altitudinal Gradients
Source: Plant Environ Interact. 2025 Sep 8;6(5):e70083. doi: 10.1002/pei3.70083 (PMC12415870; doi:10.1002/pei3.70083)
Supplement: Supplementary file 3 — Table S2: Gene Name and the Primers List Used in This Study Including the Accession Numbers and Gene Abbreviation. [file PEI3-6-e70083-s004.docx]

Table S2: Gene Name and the Primers List Used in This Study Including the Accession Numbers and Gene Abbreviation

| Gene Name | Abbreviation | Accession No | Left primer | Right primer |
| --- | --- | --- | --- | --- |
| 1. Ripening related protein | PaAVOe3 | M32692.1 | GGGGTTTCTTTGAGCTGGTG | AAGAAGGTGCTCTCCCAGTC |
| 1. ethylene response sensor 1 | paERS1 | AF500121.1 | CCAGAACTCTCCGCAAATGG | AGATGGCCCTTTCTGCTTCT |
| 1. polygalacturonase | paPolyGal | X66426.1 | CTGTGACTCCAAGGACCCAT | TCCTTCCCTCCATTCCCAAC |
| 1. beta-D-galactosidase | PaGAL4 | AB252829.1 | ATGTGGGAAGGTCTCATGCA | TAAGGCCCAATCCGCAAATG |
| 1. ethylene insensitive 3 | paEIN3 | EU586509.1 | CGAAGTCTTCCCTCCATGGT | GAAATCCGGCGCCATATGTT |
| 1. glyceraldehyde-3-phosphate dehydrogenase | paG3PD | GQ122209.1 | GGACTGGAGAGGTGGAAGAG | ACTCCTCCTTGATTGCAGCT |
| 1. 1-aminocyclopropane-1-carboxylate synthase 1 | paACS1 | AF500119.1 | ATGTCTCTCCAGGGTCCTCT | CTAAAGACTTCGCTGCCACC |
| 1. actin | PaActin | GU272027.1 | GGCTGGATTTGCTGGAGATG | TGCTCACAATTCCATGCTCG |
| 1. Glyceraldehyde 3-phosphate dehydrogenase | paGAPDH | MH759770.1 | AGCTGCAATCAAGGAGGAGT | CAACGACACGAGCACTGTAG |
| 1. ethylene response sensor 1 | paERS1 | AF500121.1 | TTGCAGCCATGAAGAAGTTG | ATGGCTGTCACTTGATGCTG |
| 1. ethylene receptor | paETR | EU370699.2 | TAATCCGAAAACCCGTCTTG | GGGAGGAGGGGACTCTACTG |
| 1. ethylene insensitive 3 | paEIN3 | EU586509.1 | TATCCTTTTGGGCACGAAGT | TAGACCGCCTTACCAGGTTG |
| 1. CTR1-like protein kinase | paCTR1 | EU417962.1 | GAGAAGCGTTGGATGAGAGG | ATCGAGAGAGGCCAAAGTCA |
| 1. 1-aminocyclopropane-1-carboxylate synthase 2 | paACC1 | AF500120.1 | ATGCAACTTGGACCTCATCC | ACGAATTGTTCGTCGGAAAG |
| 1. beta-D-galactosidase | PaGAL3 | AB252828.1 | ATGGCTGTTGGTCTTGGAAC | CAAAAGCCGTAAACCAGGAA |
| 1. beta-D-galactosidase | PaGAL2 | AB252827.1 | ACTAGTTGCCCCAGTGGATG | GGGTTTCCTCCAGTTTCCTC |
| 1. alcohol dehydrogenase 3 | paADH3 | KT246106.1 | GCCTATACATGGAGCGGAAA | AACGACTTTTGCGGTCATTC |
| 1. alcohol dehydrogenase 2 | paADH2 | KT246105.1 | TGCTGTGCTAGTCGGTGTTC | CCGTCGAGCATGTACTCAAA |
| 1. alcohol dehydrogenase 1 | paADH1 | KT246104.1 | AGTTGACCGCAGCGTAGAGT | GAAGGTCCCCTTGAGAGTCC |
