# Supplementary material for: Hormonal Regulation of Avocado (Persea americana) Across Altitudinal Gradients
Source: Plant Environ Interact. 2025 Sep 8;6(5):e70083. doi: 10.1002/pei3.70083 (PMC12415870; doi:10.1002/pei3.70083)
Supplement: Supplementary file 4 — Table S3: Abscisic Acid (ABA), Jasmonic acid (JA), Salicylic acid (SA), Gibberellic acid (GA3) and Zeatin concentrations (ng/g DW) in Avocado varieties harvested from Aadbel, Abbasiyeh, Ansar, Nmeiriyeh and Qloud El Barqa locations in Lebanon. While comparing phytohormones in different locations for the same variety, means with the same letter are not significantly different from each other (p‐value > 0.05). [file PEI3-6-e70083-s001.docx]

***Table S3*** Abscisic Acid (ABA), Jasmonic acid (JA), Salicylic acid (SA), Gibberellic acid (GA3) and Zeatin *concentrations (*ng/g DW**)** *in Avocado varieties harvested from Aadbel, Abbasiyeh, Ansar, Nmeiriyeh and Qloud El Barqa locations in Lebanon. While comparing phytohormones in different locations for the same variety, means with the*same letter are not significantly different*from each other (P-value >0.05).*

| **Variety** | **location** | **ABA** | **JA** | **SA** | **GA3** | **Zeatin** |
| --- | --- | --- | --- | --- | --- | --- |
| **Fuerte** | Aadbel | 80.72± 0.22 a | 1.13± 0.27 b | 18.13± 4.64 a | 0.94± 0.11 c | 1.26± 0.06 b |
|  | Abbasiyeh | 30.32± 0.31 b | 1.13± 0.27 b | 4.93± 0.63 bc | 1.72± 0.19 b | 3.53± 0.14 a |
|  | Ansar | 24.73± 0.05 d | 8.06± 1.58 a | 9.19± 0.55 abc | 2.55± 0.23 b | 1.72± 0.03 b |
|  | Nmeiriyeh | 22.06± 0.22 e | 1.68± 0.21 b | 14.4± 1.42 ab | 5.65± 0.37 a | 1.21± 0.05 b |
|  | Qloud El Barka | 27.11± 0.02 c | 0.9± 0.28 b | 0.96± 0.26 c | 0.8± 0.02 c | 1.02± 0.07 b |
| **Hass** | Abbasiyeh | 27.84± 0.2 c | 8.55± 1.33 a | 16± 3.13 c | 2.21± 0.07 b | 3.94± 0 ab |
|  | Ansar | 21.62± 0.6 e | 6.04± 0.3 a | 9.46± 1.12 c | 3.51± 0.53 ab | 4.43± 0.07 a |
|  | Nmeiriyeh | 20.81± 0.32 e | 1.75± 0.05 c | 14.32± 2.38 c | 4.48± 0.14 a | 2.51± 0.07 b |
|  | Mrwaniyeh | 24.58± 0.53 d | 1.71± 0.18 c | 28.14± 8.44 b | 3.83± 0.27 ac | 1.02± 0.1 b |
|  | Beit Mallat | 45.31± 0.4 a | 3.34± 0.55 b | 7.57± 1.69 c | 4.98± 0.18 a | 3.17± 0.06 ab |
|  | Aadbel | 34.63± 0.54 b | 2.19± 0.07 bc | 63.88± 7.31 a | 2.91± 0 bc | 2.12± 0.02 b |
| **Lambhass** | Abbasiyeh | 39.82± 0.95 | 7.48± 2.32 | 7.25± 4.13 | 2.94± 0.54 | 4.48± 0.55 a |
|  | Mrwaniyeh | 38.2± 2.32 | 2.61± 0.51 | 7.9± 3.17 | 3.43± 0.6 | 3.42± 0.41 ab |
|  | Beit Mallat | 42.39± 0.06 | 1.59± 0.68 | 5.62± 2.4 | 3.3± 0.05 | 1.43± 0.06 b |
| **Pinkerton** | Abbasiyeh | 14.18± 1.22 d | 3.1± 0.08 ab | 4.64± 0.45 c | 1.11± 0.12 d | 5.68± 0.06 ab |
|  | Ansar | 17.24± 0.04 d | 5.01± 0.13 a | 11.17± 0.99 b | 4.86± 0.04 b | 3.71± 0.07 bc |
|  | Nmeiriyeh | 17.32± 0.96 d | 1.41± 0.3 b | 8.4± 2.04 bc | 9.28± 0.14 a | 6.31± 0.17 a |
|  | Qloud El Barka | 25.73± 0.53 c | 2.46± 0.24 b | 31.17± 3.42 a | 0.68± 0.06 e | 3.42± 0 bc |
|  | Mrwaniyeh | 34.63± 0.18 b | 2.73± 0.24 ab | 8.79± 0.48 bc | 2.8± 0.08 c | 2.59± 0.03 c |
|  | Aadbel | 74.42± 0.38 a | 1.96± 0.93 b | 1.81± 0.35 d | 2.16± 0.09 c | 2.88± 0.12 c |
| **Reed** | Abbasiyeh | 29.62± 1.92 b | 8.32± 3.32 | 16.88± 5.45 | 2.24± 0.12 | 2.19± 0.47 |
|  | Nmeiriyeh | 52.79± 2.47 a | 5.88± 1.42 | 13.25± 2.61 | 1.63± 0.44 | 1.49± 0.41 |
